# Cases of Dropsy

**Published:** 1847-03

**Authors:** Thomas C. Osborne

**Affiliations:** Erie, Alabama


Art. VI.—*Cases of Dropsy.* By Dr. Thomas C. Osborne. of Erie, Ala.

*Case* 1. This was a case of ascites in an unmarried female, aged 18 years, the result of acute peritonitis.

In February 1842 she was seized, after exposure to cold, in her menses, with a chilliness and pain in the head, back and abdomen. I found her laboring under intense pain about the umbilicus, which was increased by pressure, her pulse 100, quick and tense, with hurried respiration and anxious countenance. The menstrual flux continued, bowels constipated. Treatment: v. s. to faintness; cal. and jal., to be followed by ol. ric. in six hours; fomentations to the abdomen.

18th. Patient better; bowels moved; respiration attended with less pain.—Castor oil and pepper poultices.

19th. Patient worse; pulse 100 and tense; she lies on her back with her knees drawn up; pressure of the bed clothes painful; menses suppressed; bowels costive.—V. s. repeated to syncope; blister over the abdomen; cal. and jal., to be followed in six hours by ol. ric. and spts. terebinth.

21st. Better in every respect. Pulse 80, soft and full, skin moist, bowels soluble, and the evacuations bilious.—Laxatives and diet.

March 9th. The blistered surface having healed, the abdomen was observed to be swelling and the respiration impeded; pulse 90, small and hard; bowels open; urine scanty, natural in color, deposited a branny sediment, not coagulated by heat.—V. s. again, and blister reapplied; cal. and squills, 2 gr. each, three times daily; bitart. potas. and jalap every morning.

10th. The blister drew, and was attended by an extraordinary effusion of serum; the bed clothing and mattress were saturated, and the liquid had run down upon the floor.—Treatment continued.

April 14th. Patient much worse. Blister applied again, but without any good result. All expedients were tried, but still the ascites continued to progress until July, when tapping was deemed indispensable. Three gallons of serum were evacuated; the girl felt much relieved; the kidneys, having been long torpid, now began to act, and lively hopes were for a few days felt, that the patient was about to recover. But these flattering appearances soon failed, and, by the last of August, the dropsical effusion was as great as ever. I determined, as a mere experiment, to employ iodine in the case, and on the first of September gave her 10 drops of the saturated tincture, to be repeated thrice daily. Six days afterwards the menstrual flux returned; the abdomen diminished steadily in size, her strength improved, and in three months her health was restored.

*Case* 2. This was a case of ascites, resulting from the improper use of a stimulating emmenagogue—the volatile tincture of guaiacum. The subject was a negro woman, in whom suppression had existed for two or three years. She was cured of the dropsy in a short time by cal., squills, and the bandage.

*Case* 3. The third case was one of ascites and anasarca in a child, following diarrhœa. A cure was effected by cal., the bandage, and tepid bathing.

February 9th, 1847.

